# Adaptive Quadruped Balance Control for Dynamic Environments Using Maximum-Entropy Reinforcement Learning

**DOI:** 10.3390/s21175907

**Published:** 2021-09-02

**Authors:** Haoran Sun, Tingting Fu, Yuanhuai Ling, Chaoming He

**Affiliations:** School of Mechanical Engineering, Southwest Jiaotong University, Chengdu 610031, China; sunhaoran@my.swjtu.edu.cn (H.S.); futingting@my.swjtu.edu.cn (T.F.); lingyuanhuai@my.swjtu.edu.cn (Y.L.)

**Keywords:** quadruped robot, multi-contact balance control, reinforcement learning (RL), artificial neural networks (ANN), soft actor-critic (SAC)

## Abstract

External disturbance poses the primary threat to robot balance in dynamic environments. This paper provides a learning-based control architecture for quadrupedal self-balancing, which is adaptable to multiple unpredictable scenes of external continuous disturbance. Different from conventional methods which construct analytical models which explicitly reason the balancing process, our work utilized reinforcement learning and artificial neural network to avoid incomprehensible mathematical modeling. The control policy is composed of a neural network and a Tanh Gaussian policy, which implicitly establishes the fuzzy mapping from proprioceptive signals to action commands. During the training process, the maximum-entropy method (soft actor-critic algorithm) is employed to endow the policy with powerful exploration and generalization ability. The trained policy is validated in both simulations and realistic experiments with a customized quadruped robot. The results demonstrate that the policy can be easily transferred to the real world without elaborate configurations. Moreover, although this policy is trained in merely one specific vibration condition, it demonstrates robustness under conditions that were never encountered during training.

## 1. Introduction

Legged robots can be used as substitutes for human beings and animals for working in harsh conditions. Although bionic structures and state-of-the-art hardware provide legged robots with agility, the full implementation of these robots is hindered by a wide range of potential factors that can destabilize robots in such scenarios. For instance, robots can waggle on aerial or aquatic platforms because of wind and water waves, tremble in post-earthquake rescue situations due to aftershocks, and become unbalanced during planetary exploration from frequent dust storms. To counteract these external disturbances, it is essential for robots to change their distribution of contact points to regulate their trunk posture, thus ensuring good performance in these dynamic environments.

Various approaches have been proposed to achieve multi-contact balancing control for legged robots. In an early exploration of balance in legged locomotion, a relatively simple algorithm is proposed for a one-legged hopping machine based on a spring-loaded inverted pendulum (SLIP) [1,2]. This algorithm decomposed the control of legged locomotion into three parts: a vertical height control part, a horizontal velocity part, and an angular attitude control part. Initialized with the one-legged system, one of the most well-known quadruped systems, Bigdog [3], performed well in self-adapting to external forces. Xu et al. [4] combined a SLIP with compliant control in terms of posture, allowing quadruped robots to reduce the effects of disturbances. In [4], inverse dynamics and Raibert’s balance controller [5] were employed to predict the desired torque of joints. Stephens and Atkeson [6] proposed a dynamic balance force controller to determine full-body joint torques based on the desired motion of the center of mass (CoM) through inverse kinematics. This approach controls the motion of the CoM and the angular momentum of the robot by computing suitable contact forces with a quadratic optimization problem. The mapping of the contact forces to the joint torques is solved considering the multibody dynamics of the system.

Khorram and Moosavian [7] proposed a controller for quadruped robots to restore the robot equilibrium in the standing phase when exerting external pushes. The method developed a full-dynamics model, with constraints of the stability, friction and saturation constraints to derive the desired forces/torques which can achieve body balance. Din et al. [8,9] presented a control method that estimates the external forces applied to legs to help quadruped robots maintain balance. In this method, a sliding-mode controller was proposed to track a desired gait with high precision in fast varying external disturbances, to calculate the optimized accelerations of the leg joints of the robot.

Another approach for realizing dynamic stabilization is whole-body control (WBC), which casts the locomotion controller as an optimization problem. WBC methods, which exploit all degrees of freedom (DoFs) for legged robots, spread the desired motion tasks globally to all the joints [10] by incorporating full dynamics. Through a passive WBC approach, Fahmi et al. [10] considered the full robot rigid body dynamics and achieved dynamic locomotion while compliantly balancing the quadruped robot’s trunk. Henze et al. [11] presented another WBC controller, which needed to solve an optimization problem for distributing a CoM wrench to the end effectors while considering constraints for the unilaterality, friction and position of the center of pressure.

Despite the fact that these approaches solved the balance control of legged robots to some degree, these controllers which are based on analytical models have strong sensitivity regarding parameters, and require considerable formulation derivation and tedious hand-tuning in the design process. When implemented on physical robots, these methods also need to address random noise and delays in data transmission due to hardware issues. In addition, due to the high specificity, those models need to be redesigned if the size or structure of the robot changes, and the analysis process must be repeated, which calls for additional design delays. Moreover, the difficulty of designing the controller increases dramatically for robots with complex structures, which requires extensive engineering expertise.

Since conventional controllers must infer ideal actions through analytical models designed by prior knowledge on kinematics and dynamics, intuitive actions for human beings and animals, even self-balancing and walking, are regarded as reasoning processes for robots. In recent years, a more direct approach, known as the data-driven method, was developed for achieving effective robotic control.

Data-driven methods, such as deep reinforcement learning (deep RL), have been demonstrated as promising methods to overcome the limitations of prior model-based approaches and develop effective motor skills for robots. Through deep RL, control policies are represented as deep neural networks (DNNs), which exploit the strong fitting ability of DNNs to avoid deriving dozens of kinematic and dynamic formulas. Moreover, the parameters of DNNs are optimized automatically by interacting with the environment iteratively through an RL framework, thus avoiding the hand-tuning necessary in most conventional methods.

A number of works have implemented deep RL on robot training in simulations, thus providing animated characters with remarkable motor skills [12,13,14,15]. Peng et al. [12] trained control policies for multiple simulated robots to learn highly dynamic skills by imitating reference motion capture clips. The motions produced by the training process were natural and consistent with those captured in the original data. Tsounis et al. [14] trained a two-layer perceptron to realize terrain-aware locomotion for quadruped robots, showing high performance in the problem of legged locomotion on non-flat terrain. Hess et al. [15] found that diverse environmental contexts can be helpful to learn complex behaviors when training locomotion policies for several simulated bodies.

In physical systems, some works have managed to realize the sim-real transfer of trained policies [16,17,18]. Hwangbo et al. [16] deployed a DNN-based controller that was trained by an RL algorithm called trust region policy optimization (TRPO), on the quadruped system ANYmal, achieving multiple gaits on flat ground. This work constructed two neural networks—an “actuator net” and a “policy net”—to represent the relationship between actions and torques and that between observations and actions to bridge the reality gap. A similar control policy presented in [17] was also learned in a physics simulator and then implemented on real robots. To narrow the reality gap, the physics simulator was improved by developing an accurate actuator model and simulating latency, and robust policies were learned by randomizing the physical environments as well as adding perturbations. Lee et al. [18] trained a controller for legged locomotion over challenge terrains by RL in simulations and indicated its robustness in real-world conditions that were never encountered during training in simulation.

However, current research on RL in real-world legged robots has mainly focused on performance under static environments, such as flat ground [16] and challenging terrains [18], while dynamic environments are also a common condition for robots when they are conducting tasks under earthquakes and storms. Unlike static environments, dynamic environments always cause various disturbances for robots. A recent work [19] observed that RL can be used to design the control algorithm for a quadruped robot to maintain balance in an unstable environment. In [19], RL was used to optimize a table-based deterministic policy in the finite discrete state and action spaces according to kinematic equations, i.e., the optimal actions were selected from 8 alternative actions through kinematic formulations when the quadruped system reached new states. Then an artificial neural network (ANN) is trained using the obtained pairs of states and actions through supervised learning to approximate the table-based policy, forming a continuous policy. However, there are still some problems to be further discussed. Firstly, although RL and ANN were employed, this method still highly relied on kinematic equations, which led to similar complexity of conventional methods. Another problem is that the exploration ability of RL was reduced due to the use of the deterministic policy and discrete spaces of states and actions, which easily made the policy fall into local optimum. Thirdly, the kinematic equations merely considered the angles of joints and torso, which led to the absence of some vital physical factors (e.g., gravity, force, and velocities and torques of actuators) in RL process, making the obtained policy less robust or even ineffective in different conditions.

This paper proposes a convenient and adaptable approach to construct a self-balancing controller for quadruped robots. Our method employs RL and ANN for policy design, however, the design concept and process are thoroughly different from those in [19]. This work aims at learning self-balancing control policy during interaction with a simulated dynamic environment, and transferring the obtained policy to real robots, which abandons the construction of kinematic equations to simplify the design process and enhance the adaptivity of control policy. In this paper, the self-balancing task is regarded as a continuous optimization problem, which consists well with it in the real world. As it is demonstrated that a challenging suite of training scenarios can help the trained policy succeed in a wide range of cases [14,18], we design an automatic changing disturbance curriculum to appropriately enhance the level of difficulty. A parameterized stochastic policy based on ANN is directly integrated into the RL process, other than using ANN to approximate a table-based deterministic policy that has already been obtained by RL [19]. The diversity of actions and exploration ability are ensured thanks to the employment of the changing disturbance curriculum, continuous spaces, a stochastic policy and a maximum-entropy RL framework. During the interaction between the robot and simulated environment, physical factors, such as gravity, collision, force, acceleration, and velocities and torques of actuators, are naturally considered, making the policy adaptive to a wide range of multiple vibration frequencies and amplitudes.

## 2. Materials and Methods

**Figure 1:**
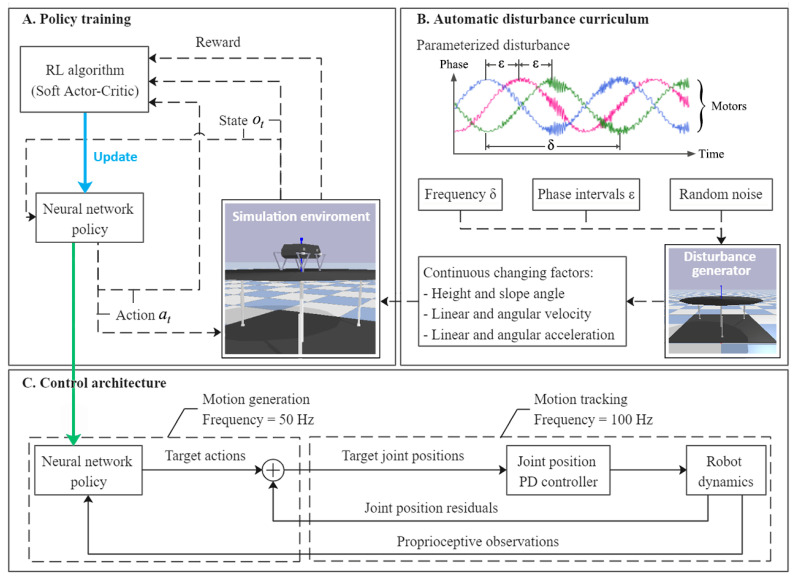
Overview of training and control architecture.

In order to endow the trained policy with strong transfer ability and robustness, this work designed a highly challenging disturbance curriculum with several continuous changing factors and random noise (see Figure 1B), and used the maximum entropy method to deeply explore appropriate actions (see Figure 1A). The training process and deployment architecture of the controller in the simulator are briefly shown in Figure 1. This section will introduce the training curriculum and training algorithm in detail.

### 2.1. Policy Training Details

The balance control of legged robots is a problem of sequential decision making. Moreover, what decision the controller makes to maintain balance in any state is not affected by previous states and actions. Therefore, the balance process is Markovian, and can be regarded as a continuous-time Markov decision process (MDP). MDP is constructed based on a pair of interactive objects, namely the agent and the environment. A typical MDP can be described as a tuple (S, A, P, r, γ) in which S represents the set of states, A the set of actions, P the state transition probability matrix, r the scalar reward function and γ the scalar discount factor. At each time step t, the agent performs an action $a_{t}\in A$ conditioned on a state $s_{t}\in S$ according to a policy $\pi \left(a|s_{t}\right)$, then the state of the environment changes to $s_{t+1}\in S$ with a reward r obtained. The accumulation of rewards r over time is called return. Finally, the policy would be automatically updated towards the maximum expected return, which can be represented as Equation (1).

(1) $$\pi^{*}=\underset{\pi }{\mathrm{argmax}}E_{\left(s_{t},a_{t}\right)∼\rho_{\pi }}\left[\sum_{k=t}^{\infty }\gamma^{k}r_{t+k}\right]$$

#### 2.1.1. Observations and Actions

In this work, actions are composed of position commands for the eight joints in both the simulation and real system, represented as $a_{t}\in {\mathbb{R}}^{8}$. The set of observations O⊆S represents the observable states, which should be directly obtained or inferred from sensor data in our method. With regard to the robot in our work, the observations $o_{t}\in O$ are specified as follows: $p_{j}\in {\mathbb{R}}^{8}$ are the angular joint positions, $v_{j}\in {\mathbb{R}}^{8}$ the angular joint velocities, $t_{j}\in {\mathbb{R}}^{8}$ the joint torques, and $q_{B}\in {\mathbb{R}}^{4}$ the quaternion representing the attitude of the robot trunk. In training and validation processes, the approaches used to obtain observations are slightly different and will be discussed later in this article. Since there is always a gap between the simulation environment and the actual robot, to increase robustness in the real environment, we add random noise with a maximum value of 5% to the observations and actions.

#### 2.1.2. Reward Function

The reward function is constructed according to the objective of this work for balancing the robot in dynamic environments. In this study, the balance control objective is defined as minimizing the tilt angle of the robot trunk under continuous external disturbance.

Therefore, the reward function in the RL training process is established as Equation (2).

(2) $$r=1-{\left\|o_{b}-{o_{b}}^{\prime }\right\|}_{2}-k\cdot {\left\|p_{b}-{p_{b}}^{\prime }\right\|}_{2}$$

where k is a ratio factor, $o_{b}$ and ${o_{b}}^{\prime }$ are the Euler angles of robot trunk relative to the world coordinate system before and after executing commanded action $a_{t}$, and $p_{b}$ and ${p_{b}}^{\prime }$ are the absolute positions of the robot trunk before and after that.

Since the termination condition is that the training episode ends when the robot falls (see details in Section 2.2), the most basic objective becomes to try not to fall after each action. In Equation (2), the constant 1 is added, which means the agent would get a score if the fall does not occur after executing commanded action. In addition, for the consideration of safety and energy consumption, position change ${\left\|p_{b}-{p_{b}}^{\prime }\right\|}_{2}$ is introduced as a penalty term to reduce unnecessary movement of the robot, with coefficient k to adjust the importance of this sub-objective. Here, k is set to 0.01.

#### 2.1.3. Policy Network

**Figure 2:**
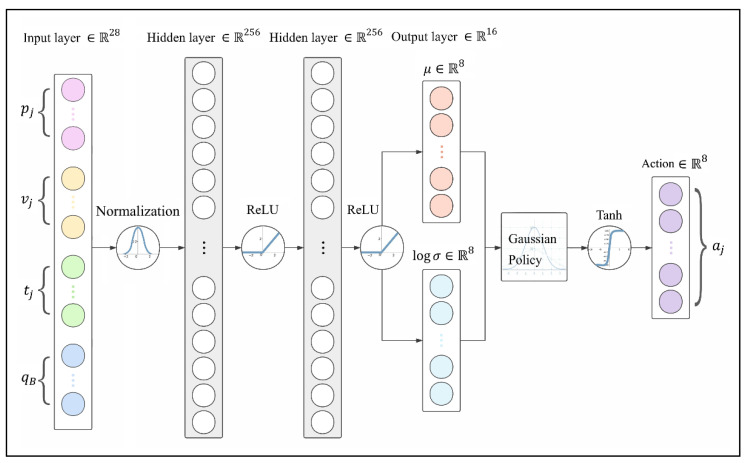
ANN-based Tanh Gaussian policy.

The balance control policy presented in this work is a combination of Tanh Gaussian policy and an artificial neural network (ANN) with two hidden layers, with 256 units each and rectified linear unit (ReLU) activation functions, as shown in Figure 2.

Observations are passed into the input layer which consists of 28 neuros, and the output layer produces 8 pairs of the mean $\mu_{j}$ and the logarithm of variance $\log\sigma_{j}$ to generate action commands according to Tanh Gaussian policy, which can be described as Equation (3). The introduction of the Tanh function compresses $a_{t}^{j}$ to make it in a bounded range, since the actions of legs are limited by the robot’s structure. In Equation (3), φ is a coefficient to scale the codomain of Tanh to the range that the motors are able to reach.

(3) $$a_{t}^{j}=\varphi \cdot \tanh x_{j}\;\mathrm{where}\;x_{j}\sim N\left(\mu_{j},\;\sigma_{j}\right)$$

#### 2.1.4. Maximum-Entropy RL Policy Training Algorithm

Since sets O and A are continuous spaces that coincide well with those in the real world, the control process can be formalized as an infinite MDP. We use a parameterized stochastic policy $\pi_{\theta }\left(a|o_{t}\right)$, which is a distribution over actions $a_{t}\in A$ conditioned on observations $o_{t}\in O$ that represents the set of observable states for the agent (e.g., data from built-in sensors of robots in our work). The optimal policy $\pi^{*}$ can be learned by automatically updating the parameter vectors $\theta \in {\mathbb{R}}^{n}$ of policy $\pi_{\theta }\left(a|o_{t}\right)$ when robots interact with the environment.

A variety of RL algorithms can be applied to such a policy optimization problem. However, there are two common deficiencies of model-free RL methods in practice: high sampling complexity and fragile convergence. The former is a problem encountered in on-policy algorithms exemplified by TRPO [20] and proximal policy optimization (PPO) [21]. In each iteration, on-policy algorithms need to re-collect enough samples under the temporal policy and completely discard the previous sampling data, which requires a large sample amount and complexity to ensure convergence. The latter is a problem that obstructs the application of off-policy algorithms exemplified by deep deterministic policy gradient (DDPG) [22] and distributed distributional deep deterministic policy gradient (D4PG) [23]. Although these algorithms reuse samples from previous iterations via replay buffers and enhance the sample efficiency, they have poor convergence and unstable performance in continuous states and action spaces and are sensitive to hyperparameters.

To alleviate these two problems, Haarnoja et al. [24] presented the soft actor–critic (SAC) algorithm which introduced the maximum entropy model to improve the randomness of actions. Equation (4) is the optimization objective of the SAC algorithm. It can be inferred from Equation (4) that, while maximizing the sum of cumulative rewards, the entropy term $H\left(\pi \left(\cdot |s_{t}\right)\right)$ deconcentrates the distribution of generated actions simultaneously, enhancing exploration ability and avoiding the dilemma of greedy sampling. Moreover, because a larger action space is learned, it is easier to make adjustments in the face of changing environments, which greatly improves the generalization ability and robustness, thus being utilized as the policy training algorithm for this work.

(4) $$\pi^{*}=\underset{\pi }{\arg\max}E_{\left(s_{t},a_{t}\right)∼\rho_{\pi }}\left[\sum_{k=t}^{\infty }\gamma^{k}r_{t+k}+\alpha H\left(\pi \left(\cdot |s_{t}\right)\right)\right]$$

### 2.2. Automatic Disturbance Curriculum

To efficiently train the control policy in a reasonable time, and to avoid damage to the physical robot caused by random actions in the exploration process, the control policy is trained only in the simulated environment, thus we need a physical simulation platform that is both accurate and fast. PyBullet [25] is a physics engine mainly used in real-time collision detection and multi-physics simulations, which is utilized by many researchers for robotic learning. A number of reinforcement learning projects, especially mobile robots, are conducted based on this engine.

**Figure 3:**
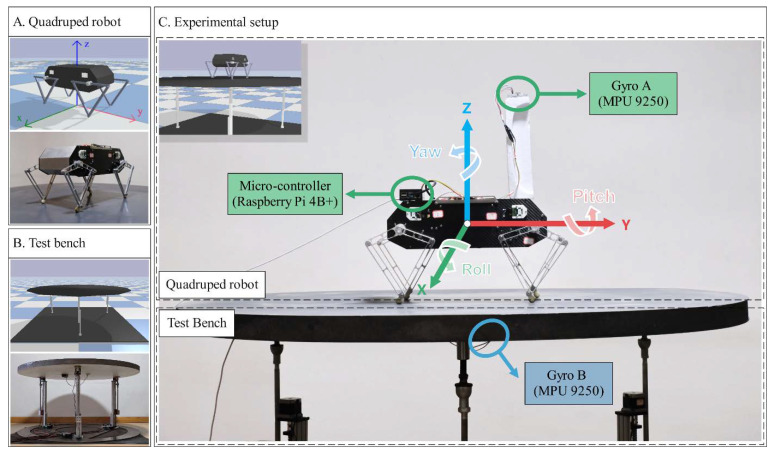
Experimental setup in simulation and real world.

The quadruped system used in our work, Stanford Doggo, is an open-source quasi-direct-drive robot comprising an unactuated base and four-leg linkages actuated by 8 motors [26]. A physical model of Stanford Doggo is constructed in the PyBullet environment, as shown in Figure 3A. The trunk of the robot is simplified into a polyhedron of uniform mass, without modeling the internal motor cables and electronic components, through which the inertial properties are estimated by the physics engine. PyBullet provides massive functions for robot simulation, so that the observations (i.e., $p_{j}$, $v_{j}$, $t_{j}$ and $q_{B}$) of the animated Doggo can be obtained directly through embedded application programming interfaces.

In terms of the structure of the bench, three actuated prismatic joints are connected to a platform through three spherical hinges, as shown in Figure 3B. During the training and verification process, we change the speed and phase interval of the three prismatic joint actuators to make the platform generate a 3-DoF vibration (displacement along the Z-axis and rotation around the *X*- and *Y*-axes). In all experiments of this study, communication between the robot and the bench is not established; that is, the disturbance generated by the bench is unpredictable for the robot, and the robot can only perceive its own postures through a gyro on the trunk and joint encoders.

The automatic disturbance curriculum is based on the aforementioned robot and test bench. In disturbance curriculum, the velocities of the three prismatic joints of the bench are set to change with time as sine curves at customized phase intervals, that is, the positions vary as cosine curves. The parametrization of disturbance makes the dynamic environment more diverse in each period and convenient to reproduce to evaluate the performance of our policy. At the same time, the acceleration and velocity of the platform and the force exerted on the robot are changing all the time, increasing the factors of disturbance, thus forming a challenging environment for the self-balancing controller.

As shown in Figure 1B, we define parameters δ and ε to briefly describe the vibration scenarios. δ is the velocity at which the phase changes (e.g., angular velocity of motors that actuated ball screws for real bench), and ε is phase intervals among three joint motors of the test bench. To monitor the tilt angle of the test bench and the quadruped robot, $\theta_{z}$ is defined to describe the angle between the *Z*-axis of the robot/bench coordinate frame and that of the global coordinate frame. $\theta_{z}$ is obtained through a spatial rotation and projection, which can be seen in Equations (5) to (8), where α, β and γ are the three components of the Euler angle of the robot trunk.

(5) $$R=\left[\begin{array}{ccc} \cos\beta \cdot \cos\gamma & \cos\beta \cdot \sin\gamma & -\sin\beta \\ -\cos\alpha \cdot \sin\gamma +\sin\alpha \cdot \sin\beta \cdot \cos\gamma & \cos\alpha \cdot \cos\gamma +\sin\alpha \cdot \sin\beta \cdot \sin\gamma & \sin\alpha \cdot \cos\beta \\ \sin\alpha \cdot \sin\gamma +\cos\alpha \cdot \sin\beta \cdot \cos\gamma & -\sin\alpha \cdot \cos\gamma +\cos\alpha \cdot \sin\beta \cdot \sin\gamma & \cos\alpha \cdot \cos\beta \end{array}\right]$$

(6) $$z_{0}={\left[\begin{array}{ccc} 0 & 0 & 1 \end{array}\right]}^{T}$$

(7) $$z_{1}=R\times z_{0}$$

(8) $$\theta_{z}=\arccos\left(\frac{z_{0}\cdot z_{1}}{{\left\|z_{0}\right\|}_{2}\times {\left\|z_{1}\right\|}_{2}}\right)$$

**Figure 4:**
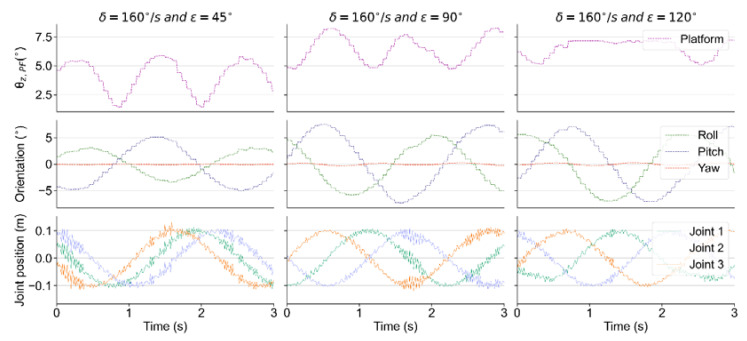
The variation in the tilt angle and Euler angle of the bench, and the displacement of the prismatic joints.

As an example, Figure 4 shows the variation in several indicators when $\delta =160^{{}^{\circ}}$/s and $\varepsilon =45^{{}^{\circ}}$, $90^{{}^{\circ}}$ and $120^{{}^{\circ}}$. Rows 1 to 3 show the time-domain variations of the tilt angle $\theta_{z}$ of the test bench, the Euler angles of the test bench, and the displacements of the three prismatic joints, respectively. Due to the definition of $\theta_{z}$, the value of $\theta_{z,PF}$ remains constant when the *Z*-axis of the test bench rotates on a conical surface around the *Z*-axis of the world coordinate system, while the disturbance generated by test bench continues, which can be inferred from rows 1 and 2 in Figure 4.

During the training process, the phase interval ε is set to $90^{{}^{\circ}}$, while the velocity δ is 100°/s. Actuated prismatic joints of the bench receive a position command about direction and amplitude at each time step, and the disturbance frequency can be adjusted via the time interval at which the commands are sent. Once the robot overturns in the dynamic environment, the ongoing training episode terminates and the next episode begins after auto-resetting. To determine whether the robot falls down, we set a termination criterion $\theta_{z,Robot}⩾\pi /2$ for each training episode.

The policy in this work is trained for 3000 epochs, and each epoch is divided into two parts: exploration (1000 steps) and evaluation (3000 steps). The exploration and evaluation processes provide a large number of samples for policy updates, and the SAC framework updates the policy based on these samples at the end of each epoch. On our computer with an E5-2430v3 CPU and a GTX 1080 GPU, the training lasts for 49.98 h, of which only approximately 9.67 h are used for calculation; the rest of the time is used for rendering and sample generation.

## 3. Verification Environment

**Figure 5:**
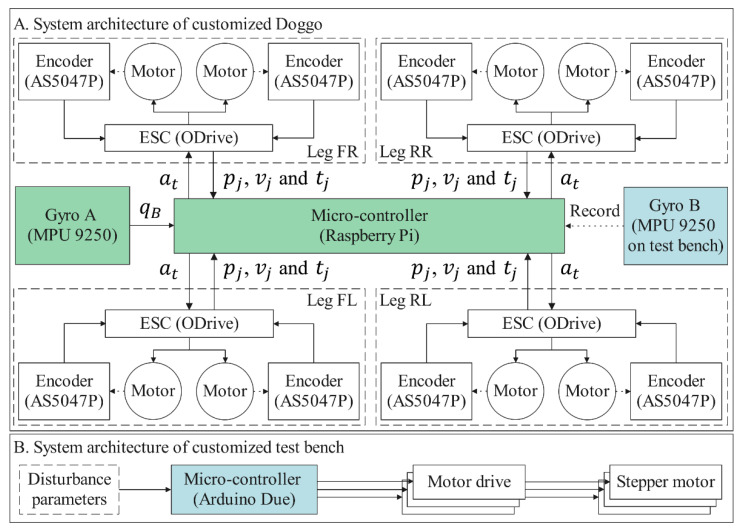
Embedded system architecture of experimental setup.

The self-balancing control policy is formed after the training process. The trained policy is deployed in simulated and real robots for validating under multiple conditions for the balance control tasks. In the simulator, the training environment can still be reused for validating, while under real conditions, our method is implemented on a customized physical Stanford Doggo, as shown in Figure 3A. Stanford Doggo achieves high performance of motor position control at low cost through the combination of eight MN5212 brushless motors (T-motor, Nanchang, China), eight AS5047P magnetic rotary position sensors (ams AG, Premstätten, Austria) and four open-source motor drive boards ODrive V3.5 48V (ODrive Robotics, San Jose, CA, USA). Compared with the original version, we use Raspberry Pi 4B+ as the microcontroller unit (MCU) to conveniently deploy the ANN-based policy with Pytorch, and use the MPU9250 gyro (TDK, Tokyo, Japan) to collect the orientation of the robot trunk. In the real-world experiment, the MPU9250 is raised to 20 cm higher from the motors, as shown in Figure 3C, because *Z*-axis data can be affected by magnetic fields. For the real Doggo, the observations (i.e., $p_{j}$, $v_{j}$, $t_{j}$ and $q_{B}$) are obtained from real sensors, in which $p_{j}$ and $q_{B}$ are collected from magnetic rotary encoders AS5047P and the gyro MPU9250, $v_{j}$ is the differential $p_{j}$, and $t_{j}$ is estimated using the current $I_{j}$ for motor j measured by ODrive through equation $t_{j}=8.27*I_{j}/KV$, where KV=340 for the motors MN5212. The embedded system architecture and data flow of the customized experimental setup are shown in Figure 5.

The customized 3-DoF test bench is also manufactured for the real experiment, on which the prismatic joints are realized by vertically installed ball screw units, as shown in Figure 3B. Under the control of an Arduino Due MCU, ball screws are actuated to enable the platform to shift along the *Z*-axis and tilt around the *X*- and *Y*-axes. The upper plate of the bench is made of lightweight materials to reduce inertia and increase flexibility. Another MPU9250 (Gyro B in Figure 3 and Figure 5) is installed on the bottom surface of this plate to record the tilt angle of the test bench, which does not interact with the control algorithm.

Although the trained policy is obtained from only one disturbance condition, various disturbance configurations are used to evaluate its performance in different conditions. In all the experiments, the robots are divided into three groups: One is the experimental group (EG), i.e., the robot using the control policy presented in this paper, the other one is the control group (CG), i.e., the robot without any control policy, and the other one is the robot using the method proposed in [19], which is called REF19 in the paper. The robot joints in CG are fixed to the initial angular position, as shown in Figure 3C.

In order to quantitatively analyze the experimental data, herein, we define indicators $\eta_{PF}$ and $\eta_{CG}$, as the balance efficiencies which represent the reduction ratios of the tilt amplitude of controlled robots compared with those of the platform of test bench (PF) and those of the uncontrolled CG, and can be calculated by Equations (9)–(12). $\eta_{PF}$ and $\eta_{CG}$ describe the balancing ability of policies from different perspectives. Moreover, if the tilt angle of external disturbance or uncontrolled robot is known, we can use the corresponding balance efficiency to estimate the tilt angle after adopting this policy to estimate whether it is worth trying to implement this policy on actual application conditions. $\theta_{z,Robot}$ can be replaced by $\theta_{z,EG}$ and $\theta_{z,REF19}$ to calculate the balance efficiencies for robots that using the policies presented in this paper and [19].

(9) $$\eta_{PF}=\left(\theta_{z,PF}-\theta_{z,robot}\right)/\theta_{z,PF}$$

(10) $$\eta_{CG}=\left(\theta_{z,CG}-\theta_{z,robot}\right)/\theta_{z,CG}$$

(11) $$\bar{\eta_{PF}}=\left(\bar{\theta_{z,PF}}-\bar{\theta_{z,robot}}\right)/\bar{\theta_{z,PF}}$$

(12) $$\bar{\eta_{CG}}=\left(\bar{\theta_{z,CG}}-\bar{\theta_{z,robot}}\right)/\bar{\theta_{z,CG}}$$

## 4. Evaluation in Simulation and Real-World Experiments

### 4.1. Simulation Results and Analysis

In this part, some experiments carried out under the conditions that the disturbance parameters are the same as the training environment, that the disturbance parameters are different from the training environment, and that the physical parameters of the robot are changed, as well as the boundary test at the end of this part.

**Figure 6:**
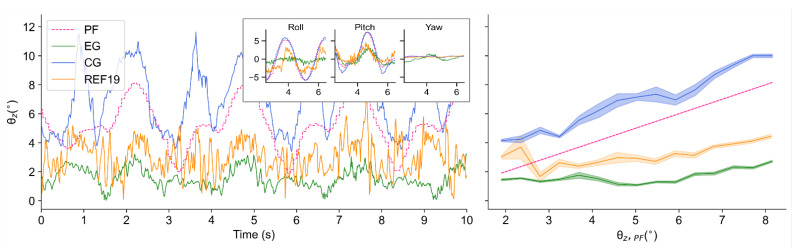
Tilt angles $\theta_{z}$ of PF, CG, EG and REF19 when $\delta =100^{{}^{\circ}}$/s and $\varepsilon =90^{{}^{\circ}}$: Time-domain variations of tilt angles (**left**); Balance performance at varying tilt angles of PF (**right**).

Firstly, this policy is verified in the same condition as the training environment ($\delta =100^{{}^{\circ}}$/s and $\varepsilon =90^{{}^{\circ}}$), performing an effective suppression on the tilt angle of *Z*-axis in the simulator. The tilt angles of PF, CG, EG and REF19 when the test bench runs at that condition are shown in Figure 6. Among the curves, the curve of PF shows how the external disturbance changes, and that of CG shows how the uncontrolled robots are affected by the disturbance, while those of EG and REF19 show how the self-balance robots behave against the changing external disturbance.

It can be seen in Figure 6 that when no balance control policy is implemented, the tilt angle of the robot is larger than that of the test bench, which is caused by inertia. After applying the policy obtained by our method, the tilt amplitude of the robot is significantly suppressed: when tested under the same conditions as the training environment ($\delta =100^{{}^{\circ}}/s$ and $\varepsilon =90^{{}^{\circ}}$), the average tilt angles are $7.21^{{}^{\circ}}$ in CG ($\bar{\theta_{z,CG}}=7.21^{{}^{\circ}}$) and $1.55^{{}^{\circ}}$ in EG ($\bar{\theta_{z,EG}}=1.55^{{}^{\circ}}$), while that of the bench is $5.25^{{}^{\circ}}$ ($\bar{\theta_{z,PF}}=5.25^{{}^{\circ}}$), which can be seen in Table 1. That is, in terms of mean values, the implementation of this policy reduces the influence of external disturbance on the balance by 71% compared with the test bench ($\bar{\eta_{PF}}=71\%$), while compared with CG, it is reduced by 79% ($\bar{\eta_{CG}}=79\%$), which can be seen in Table 2.

While applying the policy presented by [19], the tilt amplitude of the robot is about twice of ours: when tested under the same conditions, the average tilt angles are $3.02^{{}^{\circ}}$ in REF19 ($\bar{\theta_{z,REF19}}=3.02^{{}^{\circ}}$), which can also be seen in Table 1. The balance efficiencies of REF19 under such a condition are $\bar{\eta_{PF}}=42.41\%$ and $\bar{\eta_{CG}}=58.06\%$, respectively, which can be seen in Table 3.

**Figure 7:**
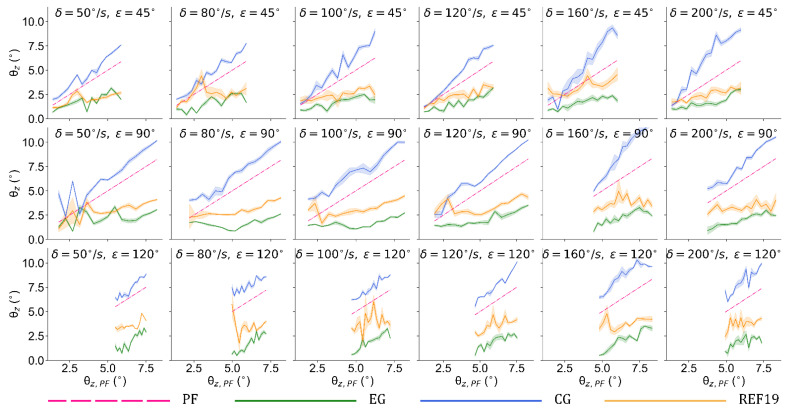
Balance performance under 18 vibration conditions.

To evaluate the policy under more disturbance conditions which are different from the training scenario, we implement the two policies in the conditions of $\delta =50^{{}^{\circ}}/s$, $80^{{}^{\circ}}/s$, $100^{{}^{\circ}}/s$, $120^{{}^{\circ}}/s$, $160^{{}^{\circ}}/s$ and $200^{{}^{\circ}}/s$. Figure 7 visualizes the balance performance under the aforementioned 18 conditions in simulation. It can be seen that the tilt angles are generally suppressed to less than 2.5° by our method when the test bench runs at tilt angles from 2° to 12°. Compared with REF19, the policy obtained by our method has an obviously better performance in almost all the scenarios.

The experimental results indicate that this policy can effectively handle disturbance in all of these conditions. The mean values of the tilt angles $\bar{\theta_{z}}$ and balance efficiencies $\bar{\eta_{PF}}$ and $\bar{\eta_{CG}}$ in these scenarios are summarized in Table 1, Table 2 and Table 3. Under 18 vibration scenarios, this control policy can keep the average tilt angle of the robot at approximately 2 degrees and offset the disturbance from the test bench by 49.37% to 71.89%, while the REF19 group 31.23% to 46.33%. Compared with CG, this approach reduces the influence of external disturbance by 60.47% to 78.53%, while the REF19 group is reduced by 45.57% to 58.14%.

**Figure 8:**
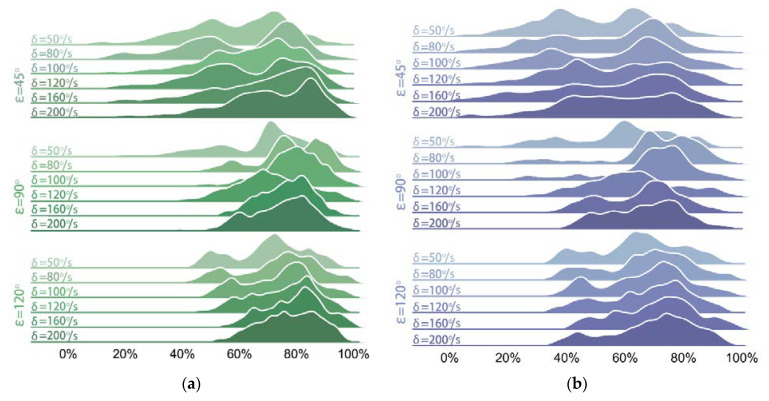
Distributions of balance efficiencies of EG under 18 conditions: (**a**) $\eta_{CG}$; (**b**) $\eta_{PF}$.

**Figure 9:**
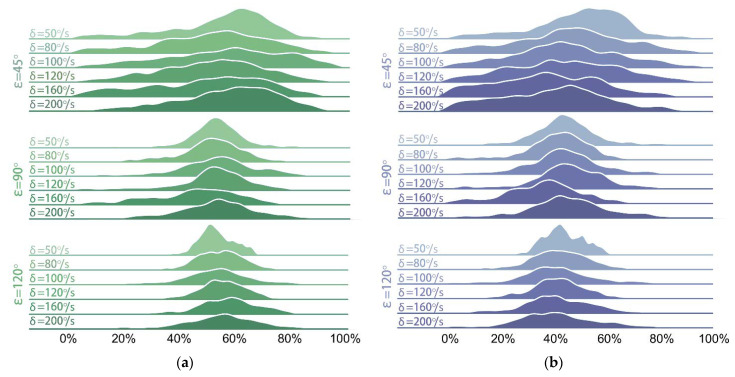
Distributions of balance efficiencies of REF19 under 18 conditions: (**a**) $\eta_{CG}$; (**b**) $\eta_{PF}$.

To further describe the performance of the trained control policy under the aforementioned 18 conditions, and to compare them with those of REF19, the distributions of the balance efficiencies relative to PF and CG are shown in Figure 8 and Figure 9.

**Figure 10:**
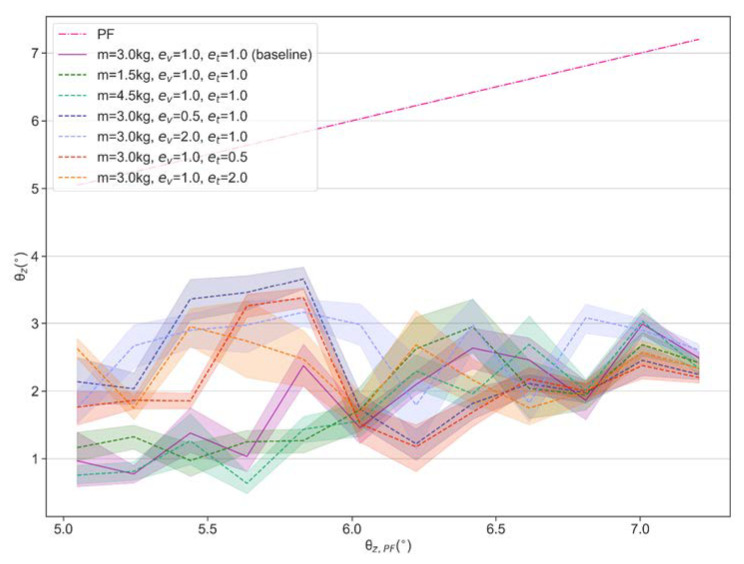
Balance performance under modified mass, velocities and torques.

In Figure 9 and Figure 10, the distributions of efficiencies of both groups are more dispersed when the phase interval is $\varepsilon =45^{{}^{\circ}}$ than those when $\varepsilon =90^{{}^{\circ}}\;\mathrm{and}\;120^{{}^{\circ}}$. A possible reason for this is that the relatively smaller phase interval results in a smaller inclination and a larger displacement of the platform along the *Z*-axis during the vibration process. Moreover, a smaller tilt angle causes more overlaps between the tilt angle curves of EG and those of the test bench, which can be seen in Figure 7, thus affecting the calculation results. Nevertheless, the lowest balance efficiencies $\bar{\eta_{PF}}$ and $\bar{\eta_{CG}}$ (when $\varepsilon =45^{{}^{\circ}}$ and $\delta =50^{{}^{\circ}}/s$) of our method still reach 49.37% and 60.47%, respectively, while those of REF19 are of its highest values 46.33% and 58.14% here.

In addition, we find that this policy provides effective balance control even if changing the body mass or scaling the observed velocities and torques through the proportional coefficients $e_{v}$ and $e_{t}$. We compare the performance under the scaled mass, velocities and torques, with a baseline which represents the performance under the initial parameter setting (m = 3 kg, $e_{v}=1.0$ and $e_{t}=1.0$). The experimental data are visualized in Figure 10. The results show that the scaled mass has little effect on the balance performance of the policy, while scaled velocity and torque have relatively larger effects when test bench runs at smaller tilt angles but similar performance with baseline at larger tilt angles. Overall, the obtained policy can effectively suppress the tilt angles to around 2° even under scaled velocity and torque conditions.

Experiments under more stringent conditions are conducted to see what kind of disturbance may cause a falling failure. According to our observation of the balancing process, three stages appear with the increase in environmental difficulty: self-balance, unstable jump, and fall, respectively. In fast (increasing at 5°/s) and mild (increasing at 1°/s) disturbance conditions, we find that at about 25°, the trained policy produces unstable performance.

**Figure 11:**
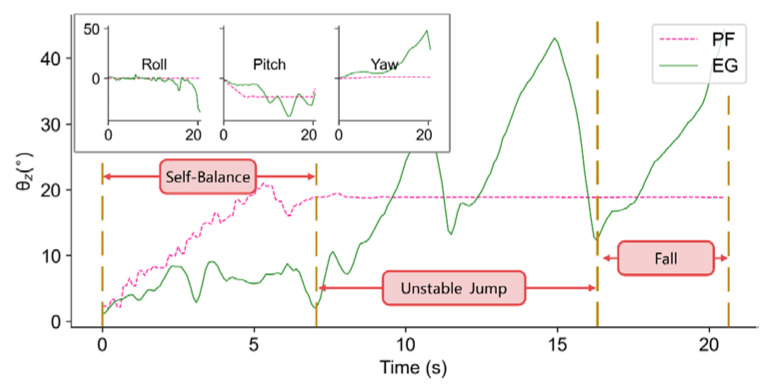
Three stages in the balance process when the platform tilts at the velocity of 5°/s.

**Figure 12:**
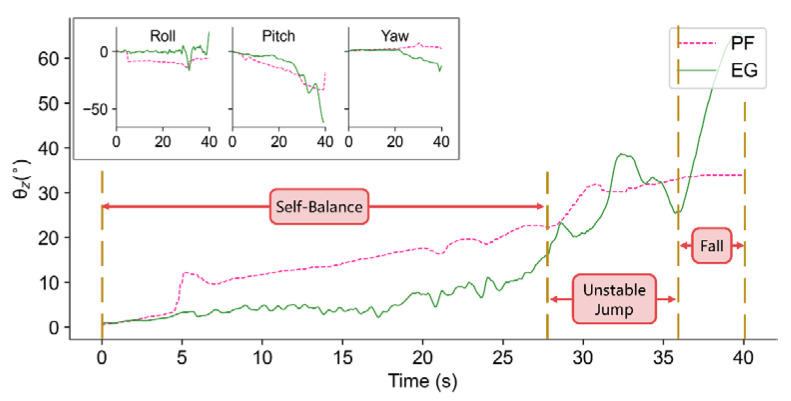
Three stages in the balance process when the platform tilts at the velocity of 1°/s.

In both cases, the policy achieves efficient balancing control until the test bench tilts to 25°. The difference is that after reaching 25° quickly, the robot shows a larger unstable jump gait with a lag of about 2 s until it falls, as shown in Figure 11. In the mild condition, the robot’s reaction is much calmer, it tilts with the test bench and behaves a mildly unstable jump, until the test bench reached about 33° when it falls, as shown in Figure 12.

### 4.2. Real-World Experiment Results and Analysis

Four episodes of EG and CG experiments from the front and lateral sides are recorded to intuitively demonstrate the balancing process. The t0 moment in the horizontal axis is the initial state of each experiment, and t1, t2, t3, and t4 represent several moments in the sequence.

**Figure 13:**
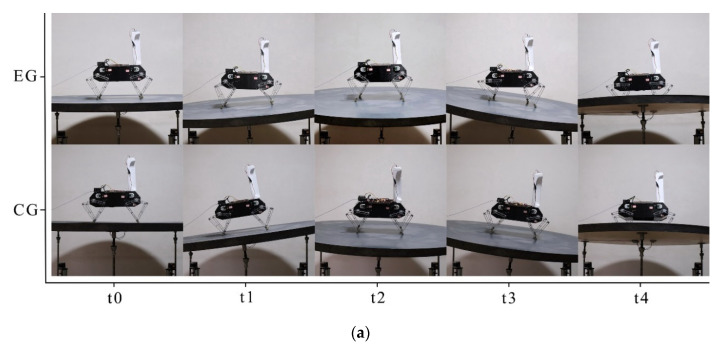

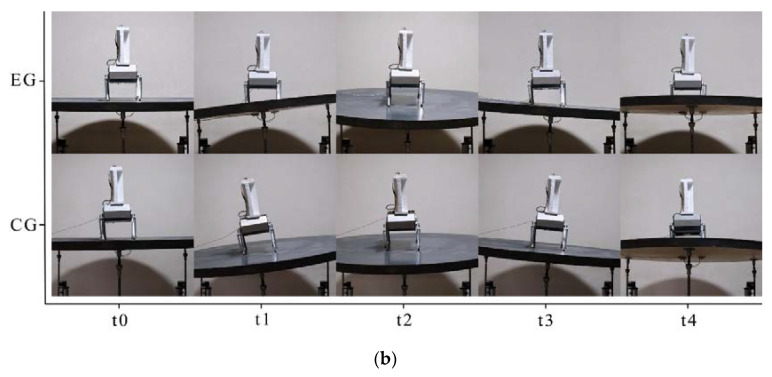
Snap-shots in real-world experiments: (**a**) Lateral view; (**b**) Front view.

In Figure 13, from the camera view, the balancing performance of this policy can be clearly seen when the test bench tilts to the left and right at moments t1 and t3. At moments t2 and t4, the test bench tilts to the front and back, whether the robot leans forward, backward or remains balanced can be inferred by whether its abdomen or back is exposed.

**Figure 14:**
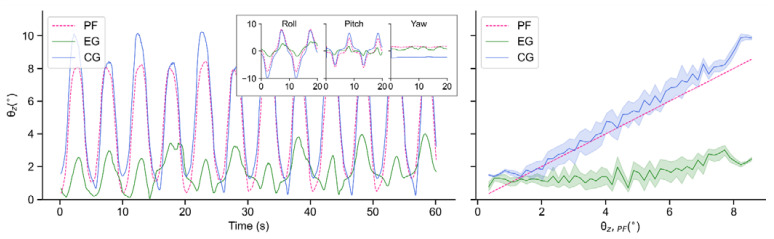
Tilt angles $\theta_{z}$ of PF, EG and CG in real-world experiments when T = 10 s: Time-domain variations of tilt angles (**left**); Balance performance at varying tilt angles of PF (**right**).

**Figure 15:**
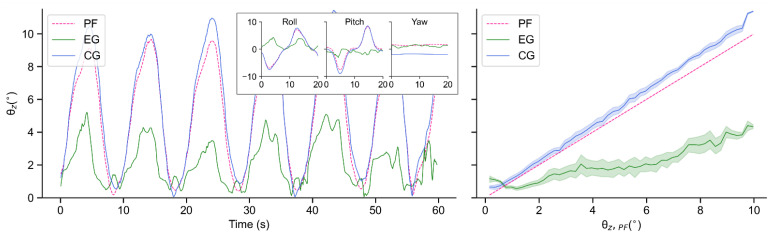
Tilt angles $\theta_{z}$ of PF, EG and CG in real-world experiments when T = 20 s: Time-domain variations of tilt angles (**left**); Balance performance at varying tilt angles of PF (**right**).

To evaluate the performance of the real robot, we collect and analyze the data of EG and CG when the motor period *T* is 10 s and 20 s, respectively. In each period, motors of the test bench run sinusoidally in accordance with a certain phase interval. However, since the tilt value $\theta_{z}$ is always a positive value, the variation period of $\theta_{z}$ is approximately half of motor period T. This is why the tilt angles of the test bench change in periods of 5 s and 10 s in Figure 14 and Figure 15, respectively.

As seen in Figure 14 and Figure 15, due to its own inertia, $\theta_{z,CG}$ is generally larger than $\theta_{z,PF}$. In terms of EG, although the robot also tilts with the test bench to a certain extent, the amplitudes are obviously decreased, which indicates an effective suppression of external disturbance. In addition, when *T* = 20 s, this proposed controller performs more smoothly than that when *T* = 10 s.

**Figure 16:**
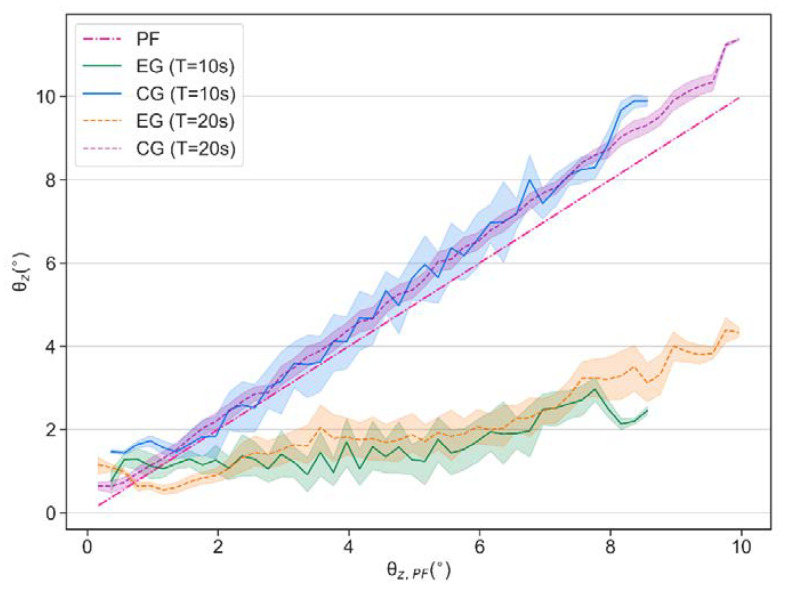
Comparison of balance performance when T = 10 s and T = 20 s in real-world experiments.

Figure 16 shows the comparison of the balance performance curves, in which it can be seen that in terms of each tilt angle $\theta_{z,PF}$ this controller demonstrates a similar balancing ability even though the vibration frequency changes, and most of the time the vibration amplitude can be suppressed to about 1.5° even though the test bench tilts to about 7°.

According to the mean values in Table 4, when T = 10 s, balance efficiencies $\bar{\eta_{PF}}$ and $\bar{\eta_{CG}}$ reach 61.89% and 66.21%. When T = 20 s, they are 57.89% and 61.83%, respectively.

**Figure 17:**
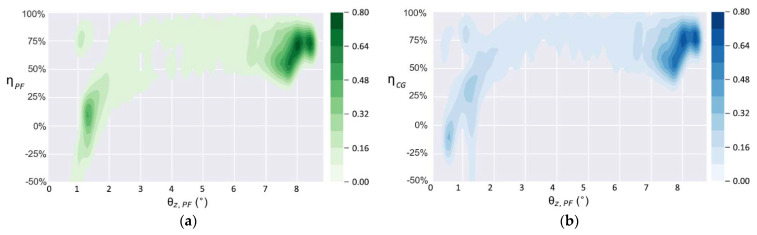
Density distribution of balance efficiencies when *T* = 10 s: (**a**) $\eta_{PF}$; (**b**) $\eta_{CG}$.

Figure 17 shows the density distribution of the balance efficiency with the tilt angle of the test bench $\theta_{z,PF}$ when working at *T* = 10 s. When $\theta_{z,PF}$ is more than 2°, the balance efficiencies $\eta_{PF}$ and $\eta_{CG}$ are mostly distributed in the range of 50% to 90% and concentrate around 75% when the test bench runs at the tilt angle of approximately 8°. The negative value of balance efficiency in the figure emerges when the $\theta_{z,Robot}$ is greater than $\theta_{z,Bench}$. As shown in Figure 17, when the test bench runs at the tilt angle of approximately 1.5°, a negative balance efficiency emerges, and the balance efficiency at this time distributes from −50% to 90%, which indicates that the controller cannot always be sensitive to a slight inclination. According to Equations (9) and (10), if the tilt angle curve of EG has a slight overlap with that of PF or CG when $\theta_{z,PF}$ or $\theta_{z,CG}$ is a small tilt angle, an exaggeratively negative balance efficiency is obtained because of the tiny denominator. As can be seen from Figure 14 to Figure 15, when such an overlap occurs, the tilt angle of the test bench and that of the robot are at a small angle of near-balance. Therefore, although the negative balance efficiency has a negative impact on the calculation of equilibrium efficiency, it has little impact on the actual performance of the self-balancing policy.

**Figure 18:**
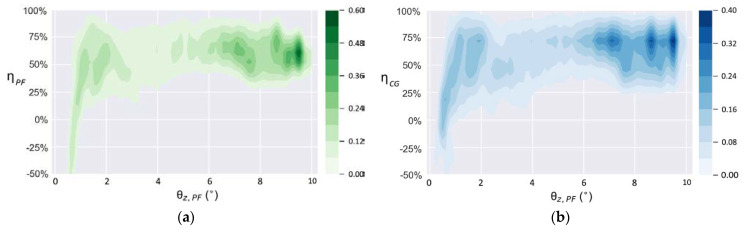
Density distribution of balance efficiencies when *T* = 20 s: (**a**) $\eta_{PF}$; (**b**) $\eta_{CG}$.

When working under the condition *T* = 20 s, the density distribution of the balance efficiency is shown in Figure 18. The most concentrated distribution is still in the area with the largest $\theta_{z,PF}$. When $\theta_{z,PF}$ reaches more than 6°, the balance efficiency concentrates at approximately 60% and 75%, respectively. However, when $\theta_{z,PF}$ decreases by almost 1°, the controller also cannot always be sensitive to such a slight inclination.

**Figure 19:**
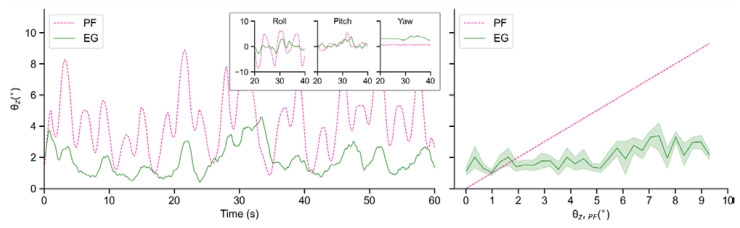
Real experiments under a relatively random disturbance condition.

The trained policy is also validated in a disturbance environment of random changing Eulerian angles, to see whether it is qualified to achieve sim-to-real transfer in more general scenarios. It can be seen in Figure 19 that, the policy in general still has a good balance performance, although there is a small overlap in the inclination curves when a sudden shock occurs. In this process, the average tilt angles of PF and EG are 4.30° and 1.84°, which means 57.27% suppression of external disturbance, which can be seen in Table 5.

## 5. Conclusions

In this study, a balance control policy is obtained through training with a maximum-entropy RL framework in a simulated dynamic environment, and validated in simulation and on a real robot, which avoids some drawbacks in the design process of conventional balance control models.

Compared with conventional methods which require tedious tuning, our method significantly simplified the design process. In our method, the learned balance control policy can be obtained by training in a simulator using an animated dynamic environment and simplified virtual robot models. There is no human involvement needed in the training process, and the engineer can carry out other work during the training. Moreover, for robots with different structures, only corresponding robot models are needed for training. These advantages greatly enhance the reusability and universality of the design process and improve the design efficiency of the balance controller.

In addition, this paper establishes an end-to-end implicit mapping of robot proprioception to action commands and proposes an ANN-based balance controller. Conventional balance control models generate action commands through the mathematical models constructed by engineers after analyzing the structure of the robot, and complete each action based on analytical models. Those hierarchical models need to be executed sequentially. In contrast, our policy can be deployed to parallel computing architecture, such as the tensor processing unit, which can greatly lower the computational time complexity and achieve an intuitive response speed in theory.

The proposed controller is validated in simulation and real-world experiments with the customized Stanford Doggo, respectively. The proposed balance controller effectively reduces the impact of external disturbance brought by the dynamic environment on robot balance. When tested under 18 conditions in the simulator, the policy reduces the external disturbance by 47.69% to 72.19% compared to the control group by 60.72% to 78.90% in terms of the median of tilt amplitude. When applied directly to the real robot, the effect is similar to that in the simulation environment: on the customized 3-DoF test bench, the average tilt angle can be reduced by 57.89% to 66.21% under regularly generated disturbances, and by 57.27% under a randomly generated disturbance.

However, the controller has a bounded input of 25° disturbance, and enhancing the difficulty of training curriculum might be one of the solutions to make it learn more to react in that condition. Moreover, it is not always sensitive when the test bench runs at a small tilt angle of less than 1.5°. Although at small tilt angles, both the test bench and the robot can be regarded as balance, some applications will demand higher accuracy and balance efficiency. For this reason, future studies can be concentrated on improving the balancing performance at small tilt angles by some techniques such as adding torque control and acceleration perception and improving the network structures. In addition, research on more complex tasks, such as self-balancing when walking in dynamic environments, will be conducted based on this work.

## Figures and Tables

**Table 1:** Average tilt angles $\bar{\theta_{z}}$ under 18 vibration conditions (°).

$\delta \;\left({}^{{}^{\circ}}/s\right)$	$\varepsilon =45^{{}^{\circ}}$				$\varepsilon =90^{{}^{\circ}}$				$\varepsilon =120^{{}^{\circ}}$			
	$\bar{\theta_{z,PF}}$	$\bar{\theta_{z,CG}}$	$\bar{\theta_{z,REF19}}$	$\bar{\theta_{z,EG}}$	$\bar{\theta_{z,PF}}$	$\bar{\theta_{z,CG}}$	$\bar{\theta_{z,REF19}}$	$\bar{\theta_{z,EG}}$	$\bar{\theta_{z,PF}}$	$\bar{\theta_{z,CG}}$	$\bar{\theta_{z,REF19}}$	$\bar{\theta_{z,EG}}$
50	4.11	5.27	2.21	2.08	5.45	6.87	3.20	2.32	6.94	8.08	3.69	2.53
80	4.01	5.06	2.75	1.87	5.34	6.94	3.07	1.50	6.70	8.22	3.72	2.29
100	4.01	5.13	2.52	1.78	5.25	7.21	3.02	1.55	6.59	8.07	4.08	2.27
120	3.74	4.89	2.38	1.67	5.74	7.13	3.27	2.20	6.63	8.45	3.87	2.23
160	4.03	6.10	3.17	1.80	6.38	8.86	4.25	2.25	6.69	9.02	3.83	2.04
200	3.72	5.96	2.53	1.67	6.39	8.13	3.55	2.16	6.75	8.85	3.99	2.13

**Table 2:** Average balance efficiencies $\bar{\eta_{PF}}$ and $\bar{\eta_{CG}}$ of EG under 18 vibration conditions (%).

$\delta \;\left({}^{{}^{\circ}}/s\right)$	$\varepsilon =45^{{}^{\circ}}$		$\varepsilon =90^{{}^{\circ}}$		$\varepsilon =120^{{}^{\circ}}$	
	$\bar{\eta_{PF}}$	$\bar{\eta_{CG}}$	$\bar{\eta_{PF}}$	$\bar{\eta_{CG}}$	$\bar{\eta_{PF}}$	$\bar{\eta_{CG}}$
50	49.37	60.47	57.47	66.31	63.58	68.71
80	53.49	63.15	71.89	78.38	65.85	72.17
100	55.52	65.26	70.54	78.53	65.56	71.89
120	55.32	65.85	61.72	69.19	66.36	73.61
160	55.32	70.48	64.70	74.56	69.46	77.36
200	55.18	72.03	66.21	73.45	68.48	75.88

**Table 3:** Average balance efficiencies $\bar{\eta_{PF}}$ and $\bar{\eta_{CG}}$ of REF19 under 18 vibration conditions (%).

$\delta \;\left({}^{{}^{\circ}}/s\right)$	$\varepsilon =45^{{}^{\circ}}$		$\varepsilon =90^{{}^{\circ}}$		$\varepsilon =120^{{}^{\circ}}$	
	$\bar{\eta_{PF}}$	$\bar{\eta_{CG}}$	$\bar{\eta_{PF}}$	$\bar{\eta_{CG}}$	$\bar{\eta_{PF}}$	$\bar{\eta_{CG}}$
50	46.33	58.14	41.20	53.35	46.19	54.29
80	31.32	45.57	42.45	55.72	44.41	54.69
100	37.07	50.81	42.41	58.06	38.15	49.49
120	36.30	51.28	42.96	54.08	41.60	54.18
160	31.23	47.96	33.33	51.99	42.81	57.58
200	32.02	57.57	44.46	56.35	40.92	54.94

**Table 4:** Tilt angles $\theta_{z}$ in real-world experiments (°).

	*T* = 10 s			*T* = 20 s		
	$\theta_{z,min}$	$\bar{\theta_{z}}$	$\theta_{z,max}$	$\theta_{z,min}$	$\bar{\theta_{z}}$	$\theta_{z,max}$
PF	0.34	4.54	8.56	0.17	4.94	9.96
CG	0.25	5.12	10.37	0.04	5.45	11.43
EG	0.06	1.73	4.01	0.04	2.08	4.46

**Table 5:** Tilt angles $\theta_{z}$ in real-world experiments (°).

	$\theta_{z,min}$ (°)	$\bar{\theta_{z}}$ (°)	$\theta_{z,max}$ (°)	$\bar{\eta_{PF}}$ %
PF	0.02	4.30	9.30	\
EG	0.04	1.84	5.66	57.27

## Data Availability

Not applicable.
